# Inhibition of NO_2_, PGE_2_, TNF-**α,** and *i*NOS EXpression by *Shorea robusta* L.: An Ethnomedicine Used for Anti-Inflammatory and Analgesic Activity

**DOI:** 10.1155/2012/254849

**Published:** 2012-05-10

**Authors:** Chattopadhyay Debprasad, Mukherjee Hemanta, Bag Paromita, Ojha Durbadal, Konreddy Ananda Kumar, Dutta Shanta, Haldar Pallab Kumar, Chatterjee Tapan, Sharon Ashoke, Chakraborti Sekhar

**Affiliations:** ^1^ICMR Virus Unit, ID and BG Hospital, GB 4, First Floor, 57 Dr. Suresh C Banerjee Road, Beliaghata, Kolkata 700010, India; ^2^Department of Applied Chemistry, Birla Institute of Technology, Mesra, Ranchi 835215, India; ^3^Division of Microbiology, National Institute of Cholera and Enteric Diseases, Kolkata 700010, India; ^4^Division of Pharmacology, Department of Pharmaceutical Technology, Jadavpur University, Kolkata 700032, India

## Abstract

This paper is an attempt to evaluate the anti-inflammatory and analgesic activities and the possible mechanism of action of tender leaf extracts of *Shorea robusta*, traditionally used in ailments related to inflammation. The acetic-acid-induced writhing and tail flick tests were carried out for analgesic activity, while the anti-inflammatory activity was evaluated in carrageenan-and dextran- induced paw edema and cotton-pellet-induced granuloma model. The acetic-acid-induced vascular permeability, erythrocyte membrane stabilization, release of proinflammatory mediators (nitric oxide and prostaglandin E_2_), and cytokines (tumor necrosis factor-*α*, and interleukins-1*β* and -6) from lipopolysaccharide-stimulated human monocytic cell lines were assessed to understand the mechanism of action. The results revealed that both aqueous and methanol extract (400 mg/kg) caused significant reduction of writhing and tail flick, paw edema, granuloma tissue formation (*P* < 0.01), vascular permeability, and membrane stabilization. Interestingly, the aqueous extract at 40 *μ*g/mL significantly inhibited the production of NO and release of PGE_2_, TNF-*α*, IL-1*β*, and IL-6. Chemically the extract contains flavonoids and triterpenes and toxicity study showed that the extract is safe. Thus, our study validated the scientific rationale of ethnomedicinal use of *S. robusta* and unveils its mechanism of action. However, chronic toxicological studies with active constituents are needed before its use.

## 1. Introduction

Inflammation is a complex biological response of vascular tissues to harmful stimuli as well as a protective attempt to remove the stimuli and initiate the healing process. Inflammation has been classified as *acute* or *chronic*. *Acute inflammation* is the initial response of our body to the harmful stimuli, achieved by the increased movement of plasma and granulocytes from blood to the injured tissues [1]. A cascade of biochemical events involving the vascular system, immune system, and various cells of the injured tissue propagates and matures the response [2]. The affected cells are then activated to release several mediators (eicosanoids, cytokines, chemokines) at the site, which elicit the inflammatory response from acute to the chronic phase. In prolonged or *chronic inflammation* a progressive shift of injured cells occurs at site and caused simultaneous destruction and healing of the injured tissues [3], during Which the release of cyclooxigenase (COX)-mediated prostaglandins leads to pain, oedema, and fever. Thus, COX inhibitors are used as antiinflammatory drugs. However, many COX inhibitors produce serious adverse effects [4] and conventional nonsteroidal antiinflammatory drugs are unsuitable for the management of chronic and silent inflammations. Moreover, most of the modern antiinflammatory and analgesic drugs are synthetic, costly, and have several side effects like nephrotoxicity, respiratory problem, constipation, physical dependence, and gastrointestinal irritation in long run. It is therefore essential to search for cost effective antiinflammatory agents with low toxicity and better tolerance from ethnomedicinal source. As the ethnomedicinal plants, in particular, are an important source of drugs and candidate therapeutics [5, 6], their scientific evaluation may provide new drug molecule to combat long-term toxicity and cost.

The Indian ethnomedicine* Shorea robusta *L. (Dipterocarpaceae), popularly known as Sal or Shal, is widely used in Ayurveda and Unani medicine. The resin is used as astringent and detergent, in diarrhoea, dysentery, and gonorrhoea [7]; With Bee wax its act as an ointment base for foot cracks, psoriasis, wounds, ulcers, burns, chronic skin diseases, and ear and eye troubles [8]; while seeds are used for pus forming wounds [9]. A combination of oleoresin with cow ghee is claimed to control burning sensation of haemorrhoids, pain, and swelling [10]. A recent study with methanol extract of mature leaves reported anti-inflammatory and antinociceptive activity [11]. However, till date there is no consistent scientific evidence of those claims with aqueous extract used in ethnomedicinal practice, and its *in vitro* and *in vivo* mechanism of action. Therefore, for the first time, we have evaluated the effect of both aqueous and methanol extracts of *S. robusta* young tender leaves in several *in vivo* and *in vitro* models. The generation of proinflammatory mediators (prostaglandins and nitric oxide) and release of proinflammatory cytokine (TNF-*α*, IL-1*β*, and IL-6) was also studied as markers, to understand the possible mechanism of action of this ethnomedicine used in traditional health care.

## 2. Materials and Methods

### 2.1. Plant Material

The young tender leaves of *S. robusta* L. were collected in April, August and December 2008 and 2009, from the nearby forest of tribal area to rule out possible seasonal variation of chemical content of the specimen. The identification and authentication was done by a Taxonomist of the Botanical Survey of India, Shibpur, Howrah, and voucher specimen (Herbarium No. 07/08/17775) has been deposited at the Herbarium and at the host Institute.

### 2.2. Preparation of Samples and Studies on Their Physicochemical Properties

Following strict standards, the collected part was washed thoroughly, dried in shade, pulverized by a mechanical grinder, and passed through 40-mesh sieve to get the fine powder. The physicochemical characters (total ash, acid insoluble ash, and water content) and the behaviour of powdered sample dissolved in different chemicals and exposed to visible and UV (312 nm) light [12] were studied for constant quality and better yield [13].

### 2.3. Extraction and Physicochemical Standardization of Extracts

Five hundred grams of powdered young leaves was extracted with distilled water (2.5 L) and 95% methanol (2 L) separately, with maceration upto 48–72 h at room temperature [14]. The extract was repeatedly filtered and centrifuged (800 ×*g* for 10 min) to remove the impurities. The collected aqueous extract was lyophilized, while the methanol extract was evaporated to dryness under reduced pressure at 40–45°C to yield crude extract (55.5 g). The % rendement or yield (w/w) of the extracts was determined by standard formula: % yield (w/w) = fixed weights of the extract/Sample weight ×100.

### 2.4. Isolation of Fractions from Aqueous Extract

The aforementioned crude extract (50 g) was subjected to phytochemical group tests of tannin (with 10% potassium dichromate/lead acetate/5% ferric chloride), reducing sugar (Benedict's and Fehling's tests), steroids (Libermann-Burchard test), terpenoids (Salkowski test), flavonoids (extract was hydrolyzed with 10% sulphuric acid, extracted with diethyl ether and divided into three parts to test with sodium carbonate, sodium hydroxide and ammonium solution) and others, following standard methods [15, 16]. The aqueous extract was then extracted with ethyl acetate (7 × 1 L) and concentrated under reduced pressure to yield a dark brown liquid mass of 1.5 g. The residual material was purified on Silica gel Column using hexane and ethyl acetate as eluent to collect six distinct fractions (F1 to F6), which were monitored by TLC [17]. The yield of fractions 1 to 6 was: 40 mg, 30 mg, 20 mg, 60 mg, 80 mg, and 250 mg, respectively.

For chromatography, the precoated HPTLC silica gel plate (60 F_254_ of 0.2 mm thickness and 20 × 10 cm size, Merck KGaA, Germany) was used. One mg/mL stock solutions of extracts were prepared in water and methanol separately. Then 100 *μ*L of each isolated fraction (1–6) was loaded on HPTLC plates at 20 mm distance, using a Linomat IV spotter (Camag, Pvt. Ltd., Switzerland). Plates were dried and developed by ethylacetate : methanol (9 : 1) solvent system, and further dried to observe under visible and UV (254 nm) light, and scanned by a Camag Scanner III (Switzerland).

The significant biological activity demonstrated by the aqueous extract intended us to further investigate the presence of plausible phytoconstituents, which may be responsible for such potential activities. Therefore, the water extracts were further subjected for purification and chromatographic fractionation.

### 2.5. Animals

Healthy Swiss albino male mice (18–20 g), and adult male Wistar rats (150–180 g) were housed in the animal house facility of the Department of Pharmaceutical Technology, Jadavpur University, Kolkata, and maintained (23 ± 4°C, relative humidity 60–70%) on a standard diet with water *ad libitum*. The animals were acclimatized for two weeks before the experiments. All animal experiments were carried out in accordance with the approval (APRO/69/20/08/09; Dated 20-08-2009) and guidelines of the Institutional Animal Ethics Committee.

### 2.6. Chemicals and Drugs

Dimethylsulfoxide (DMSO), carrageenan, phorbol-12-myristate-13-acetate (PMA), dexamethasone, NS-398 (N-[2-(cyclohexyloxy)-4-nitrophenyl]-methanesulfonamide), and lipopolysaccharide (LPS) of *E. coli *026:B6 were purchased from Sigma-Aldrich (St. Louis, Mo, USA). The L-NIL (N6-(-iminoethyl)-L-lysine, dihydrochloride) was obtained from Santa Cruz Biotechnology, Inc., USA; while RPMI 1640, fetal bovine serum (FBS), penicillin and streptomycin, trypsin-EDTA, and Tris-buffer were purchased from Gibco-BRL (Karlsruhe, Germany). The commercial enzyme linked immunosorbent assay (ELISA) kit for TNF-*α* and PGE2 was purchased from the BD Biosciences, New Jersey, USA, while IL-1*β* and IL-6 from the R&D System, Minneapolis, MN, USA, respectively. The Griess reagent, dextran, paracetamol, morphine sulfate, diclofenac disodium, and indomethacin were purchased from the respective manufacturers.

### 2.7. Acute Toxicity Study

The acute toxicity of *S. robusta* extracts (aqueous and methanol) was evaluated on different groups of mice and male Wister rats at increasing doses to determine the LD_50_. The animals were divided into ten groups (*n* = 6) and extracts were administered orally (*p.o*) at a dose of 0–2500 mg/kg, or by intraperitoneal (*i.p*.) injection at 0–1000 mg/kg. The animals were observed periodically (6, 12, 18 and 24 h) for symptoms of toxicity and death and then daily for next 14 days [18]. No acute toxic effects (agility, muscular tonus, tremors, convulsions, problem in breathing, water or food intake) or mortality was observed following treatment, so the procedure was repeated up to the highest dose of 3.5 gm/kg *p.o*. The dose regimen of the test extracts was selected (200 and 400 mg/kg) on the basis of the acute toxicity data.

### 2.8. Analgesic Activity

#### 2.8.1. Acetic Acid-Induced Writhing Tests

This was performed following a modified method of Koster and Anderson [19]. Briefly, Swiss albino mice of either sex (18–20 g) were separately divided into six groups of six animals each. The first group served as control; the second group was administered with paracetamol (50 mg/kg), while the third to sixth groups received aqueous and alcoholic extract of *S. robusta*, at doses of 200 and 400 mg/kg as *i.p.* injection. After 30 min of drug treatment, the animals were given 1% v/v acetic acid solution at 10 mL/kg *i.p*. immediately after 5 minutes of acetic acid administration and the numbers of writhing or stretches (a syndrome, characterized by a wave of contraction of the abdominal musculature followed by extension of hind limbs) were counted for 15 minutes. A reduction in the writhing number compared to the control group was considered as the analgesia [15]. The percentage inhibition of writhing was calculated according to the following formula: % Inhibition = *C* − *T*/*C* × 100, Where *C* is the mean number of writhes produced by the control group and *T* is the mean number of writhes produced by the test groups.

#### 2.8.2. Tail Flick Test

Swiss albino mice of either sex (18–20 g) were divided into six groups (*n* = 6). The tail of each mouse was placed on the nichrome wire of an analgesiometer (Techno Lab, Lucknow, India) and the time taken by the animal to withdraw (flick) its tail from the hot wire was taken as a reaction time. The aqueous and alcoholic extract of *S. robusta* at doses of 200 and 400 mg/kg were injected *i.p*., using Morphine sulphate (5 mg/kg) as standard drug. Analgesic activity was measured after 30 min of administration of extract and drug [15, 20] and the percentage inhibition was calculated by the aforementioned formula.

### 2.9. Antiinflammatory Activity

#### 2.9.1. Carrageenan-Induced Rat Paw Oedema (Acute Model)

Inflammation in the hind paw of Wistar albino rats was induced by the method of Winter et al. [21]. Animals were divided into six groups (*n* = 6). First four groups of animals were pretreated with the aqueous and alcoholic extract at doses of 200 and 400 mg/kg *i.p.*, 1 h prior to subplantar (right hind paw) injection of 0.1 mL of 1% (w/v) fresh carrageenan in normal saline. The 5th group serves as vehicle control, while the 6th group received diclofenac disodium (10 mg/kg) as positive control. The edema volume (linear circumference of the injected paw) was measured by a plethysmometer at 0 h and 1h interval upto 5 h after carrageenan injection [22]. The antiinflammatory activity was evaluated based on the ratio of the changes in paw diameter in treated and untreated group as per the formula: anti-inflammatory activity (%) = (1 − *D*/*C*) × 100, where *D* is the change in paw diameter in treated group and *C* is the change in paw diameter in untreated group.

#### 2.9.2. Dextran-Induced Rat Paw Oedema (Subacute Model)

The hind paw edema on the right foot of a rat was induced by subplantar injection of 0.1 mL of freshly prepared 1% dextran solution [23]. Paw volumes were measured 30 min before and after dextran injection. The treatment of extracts (test), vehicle (vehicle control), and standard drug (drug control) was the same as described for carrageenan model. The percentage inhibition of edema was calculated by the method of Kavimani et al. [24].

#### 2.9.3. Cotton-Pellet-Induced Granuloma (Chronic Model)

The rats were divided into six groups (*n* = 6) and were anaesthetized after shaving of the fur. Sterile preweighed cotton pellets (10 mg) were implanted in the axilla region of each rat through a single needle incision [25]. Aqueous and methanol extracts at 200 and 400 mg/kg were administered *i.p.* 60 min before the cotton pellet implementation to the first four groups. The fifth group served as vehicle control, and the sixth group received diclofenac disodium (10 mg/kg), for consecutive seven days from the day of beginning of implantation. On eighth day, the animals were anaesthetized; the cotton pellets were removed surgically and made free from extraneous tissues. To obtain constant weight, the pellets were incubated at 37°C for 24 h and dried at 60°C. The granuloma weight was calculated by measuring the increase in dry weight of the pellets of the treated and control groups.

### 2.10. Acetic-Acid-Induced Vascular Permeability in Mice

The vascular permeability in mice was tested by the method of Whittle [26] with modifications. Briefly, randomly selected mice, each with six animals, were divided into six groups. Group I served as vehicle control, groups II to V were treated with 200 and 400 mg/kg of aqueous and alcoholic extract, while group VI received indomethacin (10 mg/kg) orally. One hour after the treatment, 200 *μ*L of 0.2% Evan's blue in normal saline was injected through tail vein of each mouse (at 0.2 mL/20 gm body weight). Thirty minutes later, the acetic acid (0.6%) in normal saline (0.2 mL) was injected *i.p*. to each mouse. After 1 h, the mice were sacrificed and the abdomen was open to expose the entrails and washed with normal saline (5 mL) to collect the content in a test tube. The content was centrifuged and the absorbance of the supernatant was measured in a spectrophotometer at 500 nm. The vascular permeability effects were expressed as the absorbance (A) of the amount of dye leaked into the intraperitoneal cavity.

### 2.11. Membrane Stabilizing Activity

Membrane stabilizing activity of the extract was assessed by hypotonic solution-induced human erythrocyte haemolysis [27]. Whole blood was collected from a healthy volunteer (DC) in a heparinized tube and washed thrice with isotonic buffer (154 mM NaCl in 10 mM sodium phosphate buffer, pH 7.4) for 10 minutes at 3000 g. The RBC suspension (0.5 mL) mixed with 5 mL of hypotonic solution (50 mM NaCl in 10 mM PBS, pH 7.4) with or without the extract (0.15–3.0 mg/mL) or indomethacin (0.1 mg/mL) in triplicate was incubated (10 min at room temperature) and centrifuged (3000 g for 10 min), and the absorbance of the supernatant was measured at A_590_ nm. The percentage inhibition of haemolysis was calculated according to the formula: % Inhibition of haemolysis = 100 × {OD_1_ − OD_2_/OD_1_}, where OD_1_ is the Optical density of hypotonic buffered saline solution alone, and OD_2_ is the optical density of test sample in hypotonic solution.

### 2.12. *In Vitro* Assay of PGE_2_, TNF-*α*, and Nitric Oxide in LPS-Induced THP-1 Cell

Human monocytic THP-1 cells obtained from the National Centre for Cell Science, Pune, India, were grown at 37°C in RPMI 1640 containing heat inactivated FBS (10%), penicillin (100 IU/mL) and streptomycin (100 *μ*g/mL) at 37°C in 5% CO_2_ atmosphere. Cells at the exponential growth phase were trypsinized and suspended in complete medium at 5 × 10^5^ cells/mL [28]. Cell suspension (500 nm) was then activated with PMA (100 ng/mL) for 48 h to obtain transformed macrophages. Cell viability was checked by MTT assay and cytotoxicity of the extract was evaluated in presence or absence of LPS. The transformed cells were incubated with 100 *μ*L of extracts (0–100 *μ*g/mL), or dexamethasone (1 *μ*M) for TNF-*α*, COX-2 inhibitor N-[2-(cyclohexyloxy)-4-nitrophenyl]-methanesulfonamide, or NS-398 (10 *μ*M) for PGE_2_ as drug control and 0.5% DMSO as vehicle control, respectively. After 30 min of pretreatment cells were further incubated with 1 *μ*g/mL LPS for 24 h, and cell-free supernatants were collected to determine PGE_2_ and TNF-*α* level by EIA kits (BD Bioscience, USA) as per manufacturer's instructions.

For measuring nitrite accumulation, an indicator of NO synthesis, 100 *μ*L of the previous culture was mixed with 100 *μ*L of Griess reagent (equal volumes of 1% (w/v) sulfanilamide in 5% (v/v) phosphoric acid and 0.1% (w/v) naphthylethylenediamine-HCl) and incubated at room temperature for 10 min. The selective inhibitor of inducible nitric oxide synthetase N6-(1-iminoethyl)-L-lysine, dihydrochloride or L-NIL (10 *μ*M) was used as positive control. The absorbance was measured at 550 nm in microplate reader using fresh culture media as blank. The amount of nitrite in the sample was calculated from a standard curve prepared with fresh sodium nitrite [29].

### 2.13. Determination of IL-1*β* and IL-6 Production

IL-1*β* and IL-6 levels in macrophage culture media were quantified by enzyme immunoassay kits (R&D System, Minneapolis, MN, USA) according to the manufacturer's instructions.

## 3. Statistical Analysis

The results were expressed as mean, mean ± SEM (Standard Error Mean), and SD (Standard Deviation). The statistical significance was analyzed by Student's *t-*test for unpaired observations compared with the control, and the significance of difference among the various test and control group was analyzed by one-way ANNOVA followed by Dunnett's *t-*test.

## 4. Results

In this study anti-inflammatory and analgesic potential of aqueous and alcohol extracts of *S. robusta* young leaves was evaluated by different in *vivo* screening methods, along with the mechanism of action of the most active extract. To find out the mechanism of action, the generation of proinflammatory mediators like NO and PGE_2_ and release of cytokines like TNF-*α*, IL-1*β*, and IL-6 from PMA-activated and LPS-stimulated THP-1 cells were measured.

### 4.1. Physicochemical and Phytochemical Group Tests and Chromatographic Analysis

**Figure 1:**
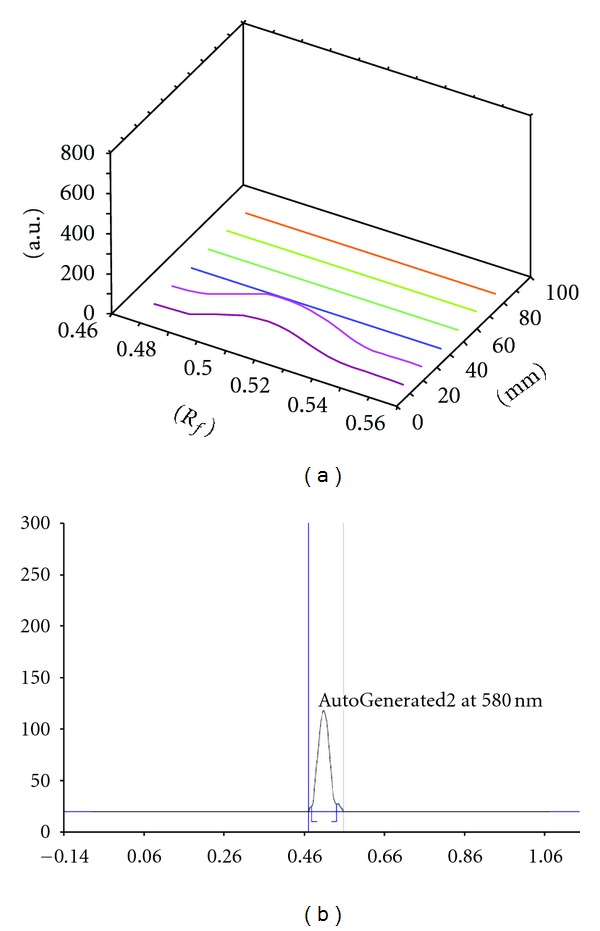
The HPTLC spectrogram (*R*_*f*_ = 0.49) of the reference compound “friedelin”, used as marker compound to evaluate the isolated fraction for comparative analysis.

**Figure 2:**
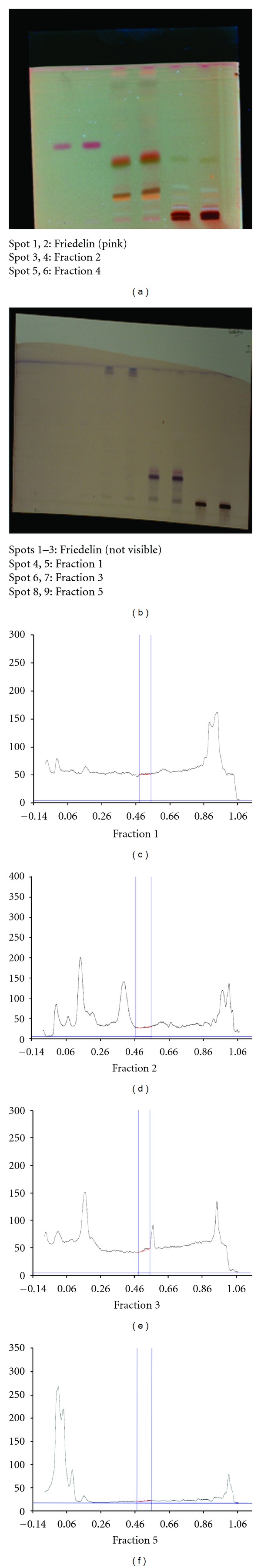
The HPTLC (a) spot 1, 2 (pink) is marker compound friedelin, spot 3, 4 is fraction 2, spot 5, 6 is fraction 4. (b) spot 1–3 is friedelin (not visible), spot 4, 5 is fraction 1, spot 6, 7 is fraction 3 and spot 8, 9 is fraction 5.

**Figure 3:**
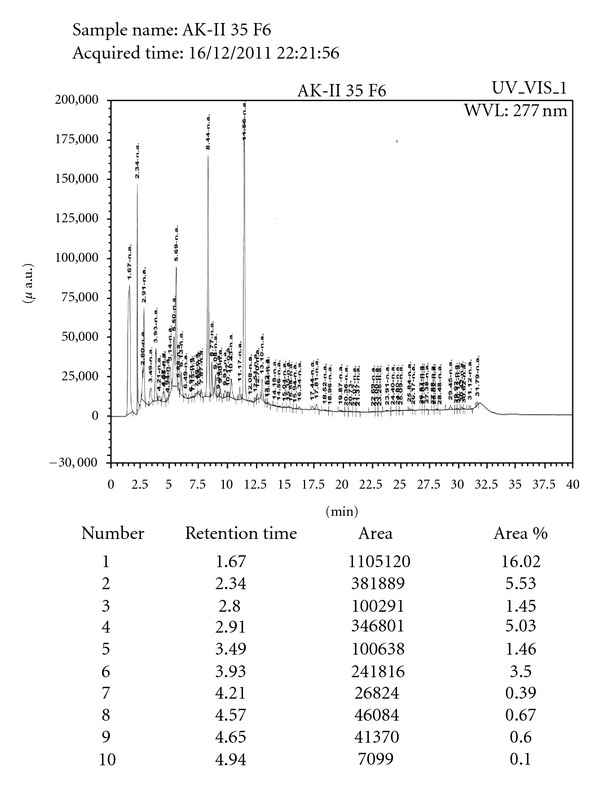
The HPLC chromatogram of fraction 6 with their retention time and area showing 4 major components.

The physicochemical study, presented in Table 1 indicated that *S. robusta* young leaves collected in August had maximum yield but minimum water (23.69%), total ash (3.55 ± 0.45) and acid insoluble ash (0.20 ± 0.3) content, compared to other samples. The fluorescence studies of the powdered samples made from the leaf collected in August showed pale yellow to yellow colour under visible and UV (312 nm) light, while with HCl, H_2_SO_4_, and HNO_3_, the colour was light to deep brown, reddish brown, and orange, respectively. However with acetic acid, chloroform, and n-hexane, the colour varied from yellow green, yellow to pale yellow, and greenish yellow and brown, respectively. The chemical group tests and preliminary HPTLC analysis (*R*_*f*_ value, percentage area, and *λ* max) of the extracts, presented in Table 2, showed that both extracts had two bands with best resolution, when ethyl acetate : methanol = 9 : 1 was used as solvent. The detail HPTLC analyses of fractions 1–6 were presented in Figures 1, 2, and 3 using friedelin as reference compound. The HPTLC analysis clearly reveals the significant purity of isolated fraction but absence of friedelin as one of the major compound in the extract.

### 4.2. Acute Toxicity Study

**Figure 4:**
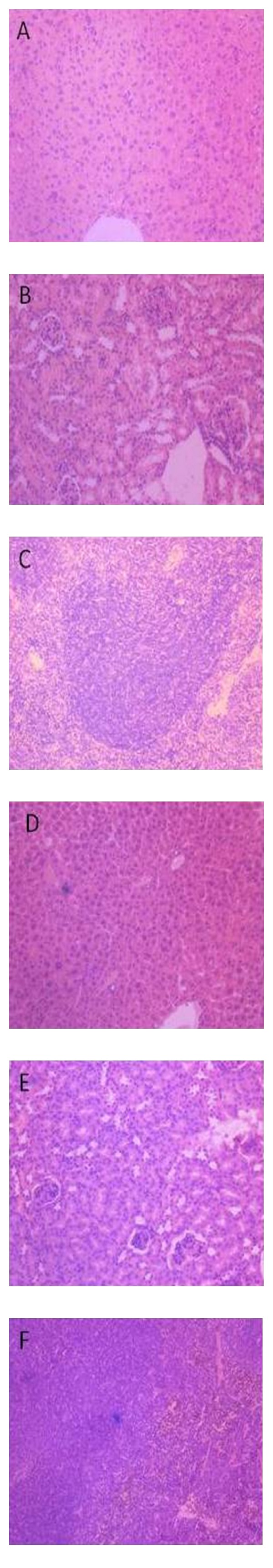
Histopathology of liver, kidney, and spleen of Swiss mice treated with aqueous extract (1200 mg/kg body weight orally). Liver (A), kidney (B), and spleen (C): control; liver (D), kidney (E), and spleen (F): treated.

Oral and *i.p. *treatment of aqueous and methanol extracts upto 14 days showed no manifestation of toxic effects (convulsion, ataxy, diarrhoea, or increased diuresis) or death in treated animals, indicating that both the extracts posses good safety profile. However, the reduced motor activity, ataxia, and hyperventilation were observed in mice, but not in rats, at oral doses of 3500 mg/kg. The LD_50_ of the orally fed methanolic and aqueous extracts of *S. robusta *was determined as 2.4 gm/kg and 2.7 gm/kg; while it was 1.2 gm/kg and 1.4 gm/kg in *i.p*, respectively. By comparing with the toxicity-rating chart [30], the extract was classified as nontoxic. Hence, the dose for further study was selected as 200 and 400 mg/kg. Further *in vivo *toxicological study for 21 days with aqueous extract upto 1200 mg/kg (*p.o*.) did not induce mortality or clinical toxicity or reveal any histopathological changes in kidney, liver and spleen (Figure 4).

### 4.3. Analgesic Activity

#### 4.3.1. Effect of Extracts on Acetic-Acid-Induced Writhing Test

The results of acetic-acid-induced writhing test with aqueous and methanol extract in mice, presented in Table 3, showed that the maximum inhibition of writhing reflexes was 60% and 63.33% at 200 mg/kg while 66.66% and 71.13% at 400 mg/kg *i.p*. of methanol and aqueous extracts, respectively. Thus, compared to the paracetamol-treated group (80% inhibition), the aqueous extracts at 400 mg/kg have equally significant (*P* < 0.001) inhibition.

#### 4.3.2. Effect of Extracts on Tail Flick Test

The results of tail flick test revealed that the aqueous extract at 400 mg/kg *i.p*. doses had reaction time of 6.6 sec (70.29% inhibition); but with methanol extract it was 7.46 sec (66.42% inhibition). However, with 200 mg/kg dose the reaction time was 7.68 sec and 8.14 sec (65.43% and 63.36% inhibition) respectively. Thus, a dose-dependent significantly (*P* < 0.001) higher effect was recorded with the aqueous extract (400 mg/kg) compared to the Morphine sulfate (5.20 sec, 76.59% inhibition) after 30 min (Table 3).

### 4.4. Antiinflammatory Activity

#### 4.4.1. Effect of Extracts on Carrageenan-Induced Rat Paw Oedema

The results of the antiinflammatory activity of the aqueous and methanol extract of *S. robusta *against carrageenan-induced paw oedema in rats showed that there was a gradual increase in the edema volume in the control group during the study period. However, both aqueous and methanol extract at 400 mg/kg *p.o.* produced a significant dose-dependent inhibition (61.90 and 65.23%, *P* > 0.001) of paw oedema, compared to Diclofenac disodium (75.71% inhibition) after 3 h of treatment (Table 4).

#### 4.4.2. Effect of Extracts on Dextran-Induced Paw Oedema

In dextran-induced paw oedema model, the maximum (69.23%) inhibition of edema swelling was noted with 400 mg/kg *p.o*. aqueous extracts, which are nearly similar to diclofenac disodium (76.22% inhibition at 10 mg/kg after 3 h), while the minimum (52.44%) inhibition was recorded with 200 mg/kg *p.o. *of methanol extract. All these data are significant (*P* < 0.0001) with respect to the control group (Table 4).

#### 4.4.3. Effect of Extracts on Cotton-Pellet-Induced Granuloma

**Figure 5:**
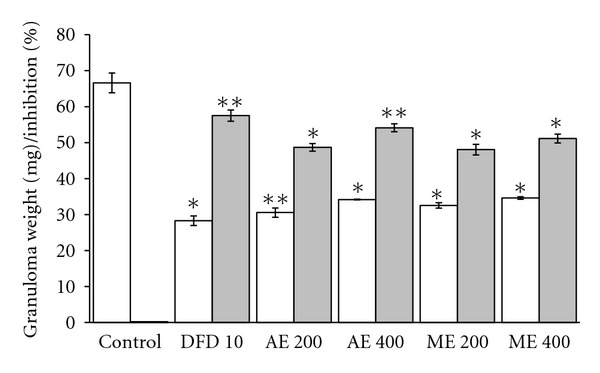
Effects of aqueous (AE) and methanol (ME) extracts on the cotton-pellet-induced tissue granulation. DFD: diclofenac disodium. White bars represent the granuloma weight while grey bars showed percentage inhibition of granuloma weight. Values are the mean ± S.E.M. of six rats. Statistical significance is represented by *P ≤ 0.05, **P ≤ 0.01, and ***P ≤ 0.001, respectively (unpaired Student's *t*-test).

The results presented in Figure 5 showed that both the extracts significantly inhibited the granuloma weight in a dose dependent manner. However, the aqueous extract (400 mg/kg *p.o*.) had significantly (*P* < 0.001) higher inhibition (54.12%) of the dry weight of granuloma compared to diclofenac disodium (57.48% at 10 mg/kg; *P* < 0.05). On the otherhand, moderate-to-high inhibitions were recorded with other doses of aqueous and methanol extract.

### 4.5. Inhibition of Acetic-Acid-Induced Vascular Permeability in Mice

**Figure 6:**
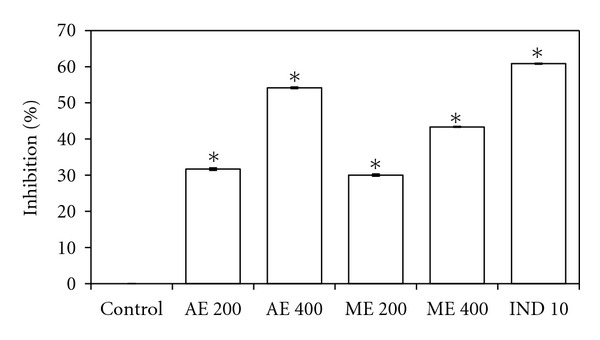
Effects of aqueous (AE) and methanol (ME) extracts on the acetic-acid-induced vascular permeability in mice. The animals were pretreated with various concentrations of the extract and indomethacin (IND). Values are the mean ± S.E.M. of six mice. Statistical significance is represented by *P ≤ 0.05 (Student's *t*-test).

Effects of aqueous and methanolic extract (200 and 400 mg/kg, *p.o*.) and indomethacin (10 mg/kg, *p.o.*) on acetic-acid-induced vascular permeability in mice, presented in Figure 6, revealed that the extract inhibited the vascular permeability by 30% to 54.16%. However, aqueous extract (400 mg/kg) significantly (*P* < 0.001) inhibited vascular permeability (54.16%) compared with vehicle control and indomethacin (60.83%) group.

### 4.6. Effects of Extracts on Membrane Stabilizing Activity

**Figure 7:**
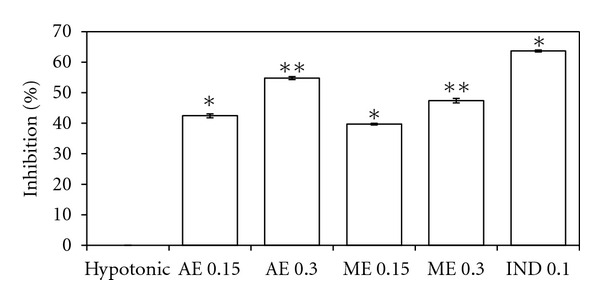
Effects of aqueous (AE) and methanol (ME) extracts on membrane stabilization of hypotonic saline-induced human RBC, in compare with indomethacin (IND). Values are the mean ± S.E.M. of six sets. Statistical significance is represented by *P ≤ 0.05, and **P ≤ 0.01 (Student's *t*-test).

The aqueous and methanolic extracts at 0.15 mg/mL doses moderately (*P* < 0.05) protect the erythrocyte membrane against lysis induced by hypotonic saline, as it inhibits haemolysis by 39.72–42.46%. However, the aqueous extract at 0.3 mg/mL and indomethacin at 0.1 mg/mL doses offered better (*P* < 0.01) protection (54.79% and 63.69%) compared to the blank (Figure 7).

### 4.7. Effects of Extracts on Cell Viability, LPS-Induced NO_2_, PGE_2_ and TNF-*α* Production

**Figure 8:**
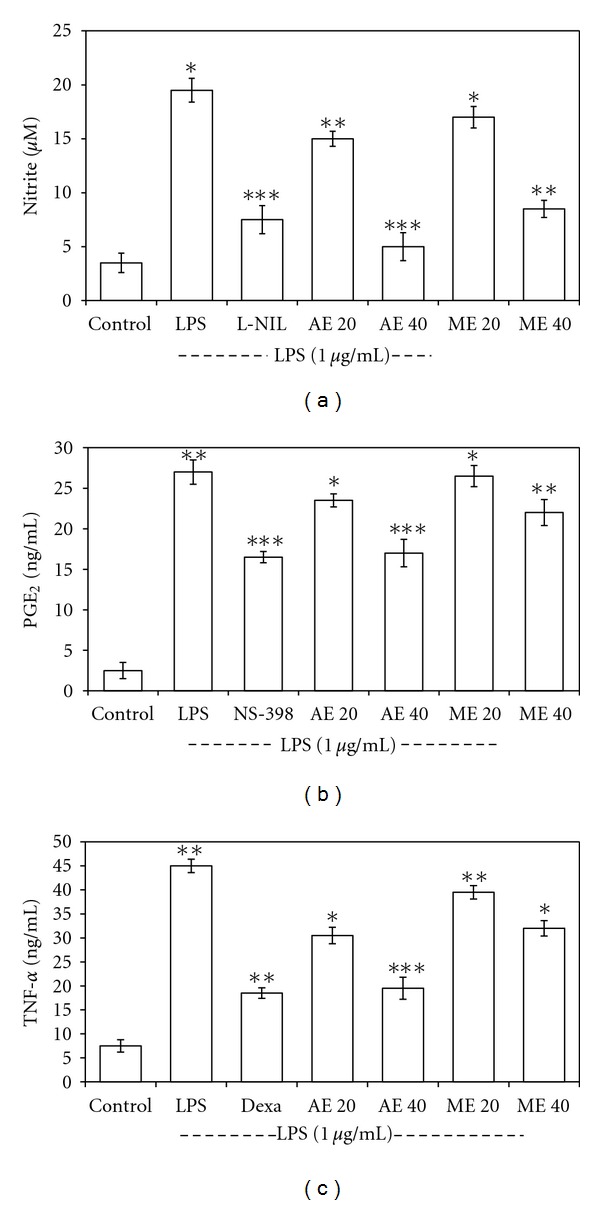
Effect of aqueous (AE) and methanol (ME) extract on nitrite (a), PGE2 (b), and THF-*α* (c) production by LPS-induced THP-1 macrophage. (a) The PMA (100 nM) activated cells were pretreated with or without various concentrations of extracts (0–100 *μ*g/mL) for 1 h and then LPS (1 *μ*g/mL) was added and incubated for 24 h. Control values are in the absence of LPS or extract while 10 *μ*M of L-NIL was used as positive control. The values are the means ± SD from three independent experiments. *P < 0.05; **P < 0.01; ***P < 0.001 versus LPS-treated group; the significance of the difference between the treated groups was evaluated by Student's *t*-test. (b) The conditions of sample treatment were identical with Figure 8(a), using 10 *μ*M of COX-2 inhibitor NS-398 as positive control. The values represent the means ± SD from three independent experiments. *P < 0.05; **P < 0.01; ***P < 0.001 versus LPS-treated group; the significance of difference between the treated group was evaluated by Student's *t*-test. (c) The conditions of sample treatment were identical with Figure 8(a), using dexamethasone (Dexa, 1 *μ*M) as positive inhibitor. The values represent the means ± SD from three independent experiments. **P < 0.01; ***P < 0.001 versus LPS-treated group; the significance of difference between the treated groups was evaluated by Student's t-test.

Cytotoxic effect of both aqueous and methanol extracts was evaluated in presence or absence of LPS and found that the cell viability was not affected by any of the extract upto 100 *μ*g/mL concentration. The effect of the extract on the production of NO, PGE2, and TNF-*α* in the supernatant of PMA-activated and then extract-treated and LPS-stimulated THP-1 cells was shown in Figures 8(a), 8(b), and 8(c). The LPS (1 *μ*g/mL) induced increased NO_2_-production was significantly suppressed by positive inhibitor L-NIL (10 *μ*M); while both the extract showed concentration-dependent inhibitory effect on LPS-induced NO production at noncytotoxic concentrations. However, the aqueous extract was most active. Additionally, the extract had no quenching effect on the Griess reagent at the concentrations used (Figure 8(a)). Furthermore, the aqueous extract significantly inhibited PGE2 (Figure 8(b)) and TNF-*α* production (Figure 8(c)) in a dose-dependent manner and is more active than its alcoholic counterpart.

### 4.8. Effect of Extract on IL-1*β* and IL-6 Production

**Figure 9:**
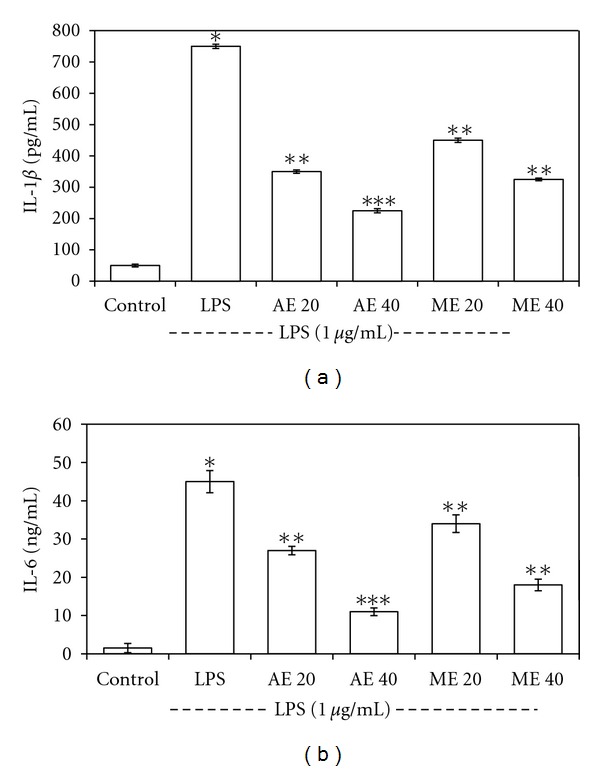
The effect of *S. robusta *extracts on LPS-induced IL-1*β* (a) and IL-6 (b) in THP-1 macrophage cells. (a) The cells were pretreated for 1 h with two selected concentrations of the extracts (20 and 40 *μ*g), and then LPS (1 *μ*g/mL) was added and incubated for 24 h. Control values were obtained in the absence of LPS or extract. The values represent the means ± SD of three independent experiments. *P < 0.05 compared with the LPS-treated group; the significance of the difference between the treated groups was evaluated using the Student's *t*-test.

To investigate the effect of extract on LPS-induced IL-1*β* and IL-6 release, we estimated the level of these cytokines in extract-treated THP-1 macrophages by enzyme immunoassay kits. The results showed that the pretreating cells with extract reduced both IL-1*β* and IL-6 production (Figures 9(a) and 9(b)) in concentration-dependent manner.

## 5. Discussion

In the Present study, we have evaluated, for the first time, the anti-inflammatory and analgesic activity of both aqueous and methanol extracts of *S. robusta *young tender leaves, used by two distinct tribes of India for ailments related to inflammation and pain, in different *in vivo *and *in vitro *model. Moreover, for the first time, the proinflammatory mediators NO and PGE2 and cytokines like TNF-*α*, IL-1*β*, IL-6 have been estimated in presence or absence of the extract to know the possible mechanism of action.

The physicochemical standardization of collected sample and extract(s) was made to achieve better yields and constant quality of the tested extracts [31]. The best quality sample was selected on the basis of physicochemical and behavioral properties, which showed that the post-rainy session sample had the maximum yield (10.1 ± 0.51) with minimum water, total ash, and acid insoluble ash content. The extraction was done by standard protocol and followed by phytochemical group tests and HPTLC analysis. The HPTLC profile of isolated fractions clearly reveals the significant purity of isolated fraction (Figures 2(a), 2(b), 2(c), 2(d), 2(e), and 2(f)). Friedelin was used as marker compound as the preliminary phytochemical tests showed the presence of triterpene and flavonoids in the crude extract. Though the isolated fraction (1–4) did not show friedelin (Figure 1), the fractions 2 and 3 (Figures 2(d) and 2(e)) show that one of the major components is very close to friedelin (parallel blue lines). This result further indicates the possibility of the presence of similar pharmacophores in Fractions 2-3, while fractions 1 and 4 have different profile and contain different nature of compounds. Thus, the HPTLC studies reveal preliminary information and warrant a detail study to establish complete structure activity relationship to demonstrated the biological significance of isolated extract. However, the HPLC profile of Fraction 6 revealed four major compounds. Earlier reports revealed that gum resin of *S. robusta* contain ursolic acid and *α*-amyrenone [32, 33], bark contains ursonic acid and oleanane [34], seed contains hopeaphenol, leucoanthocyanidin, and 3,7-dihydroxy-8-methoxyflavone7-*O*-*α*-l-rhamnopyranosyl-(1→4)-*α*-l-rhamnopyranosyl-(1→6)-*β*-d-glucopy-ranoside [35], while heartwood contains germacrene-D [10]. The isolation of *β*-amyrin, friedelin, *β*-sitosterol, pheophytin-*α*, and dihydroxyisoflavone from mature leaves was also reported [36]. However, our preliminary study indicated that the young tender leaf does not contain friedelin.

 Acute toxicity study over 14 days showed that the extract possessed good safety profile and the LD_50_ of the orally fed methanol and aqueous extracts was 2.4–2.7 gm/kg. Hence, for further study the dose was selected as 200 and 400 mg/kg. *In vivo *toxicological study with aqueous extract showed contrasting results, as the oral treatment did not induce mortality or clinical toxicity, or any histopathological changes in organs.

The acetic acid-induced writhing test indicated that the numbers of writhing movements were significantly less in the treated mice, comparable to untreated group. The effect of the extract when compared to paracetamol, suggests that the extract might have peripheral analgesic effect. Furthermore, the analgesic effect produced by the tail flick test was comparable to that of morphine-treated control, suggesting central analgesic effect.

Acute inflammatory agent's carrageenan and dextran induce inflammation through different mechanisms. Carrageenan, a standard phlogistic agent, is used to induce paw edema in animals, as it is known to release histamine, bradykinin, and serotonin (5-hydroxytryptamine) in the early phase, and prostaglandins and kinin in the late phase [37, 38], which induces protein rich exudates with neutrophil at the site of inflammation resulting in increased vascular permeability, and accumulation of fluid in tissues to form edema [39, 40]. The results of the carrageenan- induced edema test revealed that extracts at 400 mg/kg, in 3 h *p.o*, have dose-dependent inhibitory effect on edema formation in both early and late phases, comparable to diclofenac disodium. This suggests that the extract may inhibit the synthesis and/or release of those mediators, particularly the cyclooxygenase. Dextran is a high molecular weight polysaccharide that induces anaphylactic reaction characterized by extravasation and edema formation, as a consequence of liberation of histamine and serotonin [23] to the site of inflammation and the fluid accumulation through mast cell degradation [41]. Moreover, dextran can cause inflammation by activating NF-*κ*B and inducing the expression of TLR-4, and proinflammatory cytokines IL-1*β*, TNF-*α* and IL-6 in mice [42]. Thus, the inhibition of dextran mediated edema by the extract was probably due to the antihistaminic (inhibition of histamine and serotonin) effects of our extract. Furthermore, the decrease in the cotton-pellet-induced granuloma weight by the extract is due to the inhibition of proliferative phase of inflammation [22], as the inflammatory response induced by the cotton pellet can modulate the release of mediators leading to the tissue proliferation and granuloma formation [43, 44]. The acetic-acid-induced vascular permeability test is a typical capillary permeability assay, used to further confirm the antiinflammatory potential of the extract. The acetic acid is known to cause dilation of arterioles and venules and increase vascular permeability by releasing inflammatory mediators such as histamine, prostaglandins, and leukotrienes by stimulating mast cells [45]. During inflammation histamine, serotonin, and other mediators increase vascular permeability, while acetic acid causes an immediate sustained reaction [46]. Thus, the inhibition of acetic-acid-induced inflammation suggests that the extracts may effectively suppress the exudative phase of acute inflammation.

Further, the protective effect on hypotonic saline-induced RBC lysis is an index of antiinflammatory activity [47], which leads to the formation of free radical [48] that cause secondary damage through lipid peroxidation [49, 50]. Thus, the compounds with membrane-stabilizing property can protect the cell membrane against injurious substances [27, 51] by interfering the release of phospholipases that trigger the formation of inflammatory mediators [52]. Here, the observed membrane stabilizing activity suggests that the extract may inhibit the release of phospholipases and thereby the formation of inflammatory mediators.

To elucidate the *in vitro *mechanism of action, the estimation of proinflammatory mediators and cytokines, in presence or absence of the extract, was made in LPS-stimulated THP-1 macrophage cells. Macrophages play a crucial role in both nonspecific and acquired immune responses, and its activation by LPS leads to a series of responses like the production of proinflammatory cytokines (TNF-*α*, IL-1, IL-6) and activation of phospholipase A2 that produce prostaglandin and NO [53] and, thus, can be used as a model to test the potential antiinflammatory compounds [28]. Our results demonstrated that the accumulation of nitrite in the medium (due to enhanced NO production) takes place when THP-1 macrophage was exposed to LPS for hour's, and this LPS-induced NO production was significantly inhibited by the aqueous extract in a time- and concentration-dependent manner without notable cytotoxicity. This inhibition is either through the regulation of inducible nitric oxide (*i*NOS) gene expression, or its direct interference with *i*NOS activity. It is known that various anti-inflammatory drugs inhibit prostaglandins (PGs) synthesis by cyclooxygenase (COX) 1 and 2. COX-1 provides a physiologic level of PGs, while COX-2 is highly induced at inflammatory sites [54, 55]. Here, the significant inhibition of PGE2 production by the extract is probably through COX-2 gene expression in LPS-treated macrophages. However, further investigation is needed to confirm the extract action on NF*κ*B activities.

Proinflammatory cytokines (TNF-*α*, IL-1*β*, and IL-6) are known to control inflammation *in vitro *and *in vivo *[56, 57] and are probably interlinked in a cascade, produced by macrophages during inflammatory response. Moreover, the development of hyperalgesia during inflammation is probably mediated by proinflammatory cytokines [58]. Therefore, we have investigated the role of our extract in cytokine production and found that the aqueous extract significantly reduced the production of TNF-*α*, IL-1*β*, and IL-6 in dose-dependent manner. TNF-*α* plays a critical role in both acute and chronic inflammation [59] by infiltration of inflammatory cells through the adhesion of neutrophils and lymphocytes [60], and stimulates neutrophils to release cytokines (IL-1*β* and IL-6) and chemokines [61, 62]. Interaction between these mediators enhances further inflammatory reactions [63]. Thus, inhibition of TNF-*α*, IL-1*β*, and IL-6 release can reduce the severity of inflammation. The antichronic inflammatory activity of the extract observed in cotton-pellet-induced granuloma model is further supported by this study, as cellular accumulations of fluids and proinflammatory cytokines were demonstrated within the first 14 days [64–66]. Therefore, the inhibition of tissue granuloma by the extract, at least in part, is through the interference with TNF-*α*, IL-1*β*, and IL-6 release. Thus, the present study suggests that *S. robusta *young tender leaf extract, particularly the aqueous extract, inhibits LPS-induced *i*NOS and COX-2 protein expression, along with NO_2_, PGE_2_, and TNF-*α* production probably due to its terpenoids, flavonoids, or related compounds alone or in combination, as reported with other plants [67–69], and thereby it is useful in the prevention and treatment of inflammatory conditions.

## 6. Conclusion

In conclusion, the observed analgesic and anti-inflammatory activity of the aqueous extracts of *S. robusta *young leaves might be through the inhibition of leukocyte activations and reduced release of inflammatory mediators (PGE_2_, NO) and proinflammatory cytokines (TNF-*α*, IL-1, and IL-6). Therefore, our findings support the ethnomedicinal use of *S. robusta *young leaves in the management of inflammatory ailments via multilevel regulation of inflammatory reactions. Additionally its low toxicity encourages clinical trials in primary health care after subchronic and chronic toxicological studies with the active component(s).

## Figures and Tables

**Table 1:** The physicochemical properties of *S. robusta* young leave.

Sample	Sample (in gm)	Extract (in gm)	% rendement (yield)	Ash content (% w/w)	Acid insoluble ash (% w/w)	Water content (%)
*S. robusta* (Aug)	500.00	50.5	10.1 ± 0.51	3.55 ± 0.45	**0.20 ± 0.30**	**23.69 ± 12.51**
*S. robusta* (Dec)	552.80	48.9	8.84 ± 0.47	5.36 ± 0.39	0.35 ± 0.60	26.18 ± 14.47
*S. robusta* (April)	508.50	42.3	8.31 ± 0.41	6.23 ± 0.27	0.42 ± 0.17	26.56 ± 0.56

**Table 2:** HPTLC and phytochemical analysis of extracts.

Sample	Solvent system	Chromatophore (s)	*R*_*f*_	Area %	*λ* max	Phytochemical group
*S. robusta *extract	Ethyl acetate: Methanol (9 : 1)	Aqueous 1	0.78	19.33	104.3	Tannin, reducing sugar, flavonoids, Steroids
		Aqueous 2	0.87	41.93	155.4	
		Methanol 1	0.78	19.33	104.3	Tannin, flavonoids, steroids, terpenoids
		Methanol 2	0.87	41.33	155.4	
Quercetin	Ethylacetate: Methanol (9 : 1)	Aqueous Q_1_	0.79	19.34	104.5	Flavonoid
		Methanol Q_1_	0.81	19.55	105.1	

**Table 3:** Analgesic activity by acetic-acid-induced writhing and tail flick methods.

Treatment	Dose (mg/kg)	Writhing reflex (Reaction time in sec)	% Inhibition	Tail flick (Reaction time in sec)	% Inhibition
Control		15.00 ± 0.40	0	22.22 ± 0.56	0
Paracetamol	50	3 ± 0.365*	80	—	—
Morphine	5	—	—	5.20 ± 0.10*	76.59
*S. robusta* aqueous extract	200	5.5 ± 0.763*	63.33	7.68 ± 0.27*	65.43
	400	4.33 ± 0.421*	71.13	6.6 ± 0.15*	70.29
*S. robusta* methanol extract	200	6 ± 0.516*	60.00	8.14 ± 0.51*	63.36
	400	5 ± 0.365*	66.66	7.46 ± 1.00*	66.42

Results are expressed as mean ± SEM (*n* = 6), *P <0.001 compared to control (2% aqueous Tween 80 v/v).

**Table 4:** Effect of extracts on carrageenan- and dextran-induced paw edema.

Treatment	Dose (mg/kg)	Carrageenan induced paw edema	% Inhibition	Dextran induced paw edema	% Inhibition
Control	0.1 mL	2.1 ± 0.05	—	2.86 ± 0.01	—
Diclofenac disodium	10	0.51 ± 0.005	75.71	0.68 ± 0.014	76.22
Aqueous extract	200	0.97 ± 0.006	53.80	1.18 ± 0.010	58.74
	400	0.73 ± 0.008	65.23	0.88 ± 0.010	69.23
Methanol extract	200	1.08 ± 0.007	48.57	1.36 ± 0.011	52.44
	400	0.80 ± 0.009	61.90	1.07 ± 0.013	62.58

Results are expressed as mean ± SEM (*n* = 6). The significance level in comparison to control values, *P* < 0.001. Control aqueous Tween-80 solution (2% v/v).
